# Activated microglia provide a neuroprotective role by balancing glial cell-line derived neurotrophic factor and tumor necrosis factor-α secretion after subacute cerebral ischemia

**DOI:** 10.3892/ijmm.2012.1179

**Published:** 2012-11-13

**Authors:** JIANPING WANG, ZHITANG YANG, CONG LIU, YUANZHENG ZHAO, YIBING CHEN

**Affiliations:** 1 Department of Neurology, The Fifth Affiliated Hospital of Zhengzhou University, Zhengzhou, Henan 450052; 2 State Key Laboratory of Oncology in Southern China and Department of Experimental Research, Sun Yat-sen University Cancer Center, Guangzhou, Guangdong 510060, P.R. China

**Keywords:** microglia, N-(6-oxo-5,6-dihydro-phenanthridin-2-yl)-N,N-dimethylacetamide, subacute cerebral ischemia, glial cell line-derived neurotrophic factor, tumor necrosis factor-α

## Abstract

Microglia are the major immune cells in the central nervous system and play a key role in brain injury pathology. However, the role of activated microglia after subacute cerebral ischemia (SCI) remains unknown. To address this issue, we established a permanent middle cerebral artery occlusion (pMCAO) rat model and treated pMCAO rats with N-(6-oxo-5,6-dihydro-phenanthridin-2-yl)-N,N-dimethylacetamide (PJ34) (an inhibitor of microglial activation), or with vehicle alone. Finally, we determined the differences between the PJ34-and vehicle-treated rats with respect to neurological deficits, infarct volume, neuronal loss and the expression of CD11b (a marker of microglial activation), glial cell line-derived neurotrophic factor (GDNF) and tumor necrosis factor-α (TNF-α) at 1, 3 and 7 days after treatment. We found that the PJ34-treated rats had more severe neurological deficits and a larger infarct volume and exhibited a decreased CD11b expression, more neuronal loss, decreased expression of GDNF mRNA and protein but increased expression of TNF-α mRNA and protein compared with the vehicle-treated rats at 3 and 7 days after treatment. These results indicate that activated microglia provide a neuroprotective role through balancing GDNF and TNF-α expression following SCI.

## Introduction

Neuroinflammation plays an important role in the pathological process of neurological disease. Microglial activation is considered to be the hallmark of neuroinflammation (1). Previous data derived from *in vitro* or chronic neurodegenerative disease have shown that activation of microglia contributes to neuroinflammation (2) and to exacerbation of neuronal injury (3). By contrast, several recent studies have indicated that activated microglia provided a neuroprotective role and prevented neuronal loss after brain injury (4). However, the role of activated microglia in response to subacute cerebral ischemia (SCI) remains unknown. Moreover, previous findings revealed that the type of stimulus and the local microenvironment critically affect the phenotypes (5,6) and role (7) of microglia.

Since the function of activated microglia is associated with activated microenvironments, while the condition induced by SCI is significantly different from environments induced by *in vitro* stimulation (e.g. with LPS or OGP) or by chronic neurodegenerative disease, microglial role after SCI is also different from that in chronic neurodegenerative disease or *in vitro*. Hence, further *in vivo* investigations are key for exploring the effects of activated microglia after SCI.

Previous data have shown that N-(6-oxo-5,6-dihydro-phenanthridin-2-yl)-N,N-dimethylacetamide (PJ34) can inhibit activation of microglia *in vivo* by suppressing the activation of poly (ADP-ribose) polymerase-1 (PARP-1) which is a signaling pathway of the activation of microglia (8). Therefore, we selected PJ34 as the inhibitor of microglial activation.

In the present study, 156 male adult Sprague-Dawley (SD) rats were first subjected to permanent cerebral middle artery occlusion (pMCAO) and were then treated with PJ34 (an inhibitor of microglial activation), or vehicle alone. Finally, the differences between the PJ34-and vehicle-treated rats were observed with respect to neurological deficits, infarct volume, neuronal survival by NeuN staining and the expression of CD11b (the marker of microglial activation) and the mRNA and protein expressions of glial cell line-derived neurotrophic factor (GDNF) and tumor necrosis factor-α (TNF-α) at 1, 3 and 7 days following treatment. This study provides evidence that can be used to further investigate the role of activated microglia.

## Materials and methods

### Animal model of cerebral ischemia

Animal experiments were conducted in accordance with the guidelines published in the NIH Guide for the Care and Use of Laboratory Animals (US Department of Health and Human Services, Publication no. 85-23, 1985), and all efforts were made to minimize animal suffering as well as the number of animals used. One hundred and fifty six male Sprague-Dawley rats (250–300 g) were obtained from the Experimental Animal Center of Zhengzhou University. They were first subjected to pMCAO surgery and were then randomly divided into two groups with 78 rats in each group: the PJ34 group rats were treated with PJ34, and the vehicle group rats were treated with vehicle. The animals were maintained at a controlled temperature (20±2°C) and group-housed (12 h light/dark cycle) with access to food and water *ad libitum*. Infarct volume (%) was expressed as a percentage of the contralateral hemisphere.

The cerebral ischemic model was induced using a pMCAO rat model (9) under a microscope. Rats were anesthetized by inhalation of 5% isoflurane and maintained with 2% isoflurane in a mixture of 70% N_2_O and 30% O_2_. After the external carotid artery, the internal carotid artery (ICA) and the pterygopalatine artery of the ICA were exposed, a piece of monofilament nylon suture (diameter, 220 μm), with its tip rounded by gentle heating (diameter, 270±10 μm), was introduced via the lumen of the left external carotid artery stump and left ICA to embed into the left anterior cerebral artery so that the right middle cerebral artery was occluded at its origin. After the operation, rats were transferred to their cage in which the temperature was kept at 37°C until the animals woke up completely.

### Drug treatment

In the PJ34-treated group, rats were intraperitoneally injected with PJ34 at the dose of 15 mg/kg (Sigma, Mississippi; diluted in 1.0 ml sterile saline vehicle) immediately after the operation and every 12 h thereafter (8). The vehicle-treated rats were administered the same volume of saline after pMCAO.

### Measurement of neurological deficits

The measurements of neurological deficits were performed at 1, 3 and 7 days after pMCAO. All measurements were completed in a dedicated behavioral study facility during this interval to minimize environmental impact related to transfer between home cage and the testing arenas. The measured results were analyzed by individuals blinded to the experimental conditions. The battery consisted of two tests to evaluate neurological deficits: the cylinder test and the Zea Longa test. In the cylinder test, rats were placed in a clear Plexiglas cylinder (95×180 mm height) (10) and were observed for 3 min and the number of wall touches with each forelimb was scored for each rat. Normal rats touch the walls equally often with each forelimb, but hemiparetic rats touch the walls less often with the affected limb (10,11). The relative percentage of left (unimpaired) forepaw contacts was calculated as follows: (left-right)/(right+left+both) ×100%. In the Zea Longa test, neurological deficits were performed using a neurological grade score (0, no observable deficit; 1, torso flexion to the right; 2, spontaneous circling to the right; 3, leaning/falling to the right; and 4, no spontaneous movement) (12).

### Measurement of infarct volume

After the evaluation of neurological function, rats (n=6/group) were sacrificed with an overdose of sodium pentobarbital (75 mg/kg) on Day 7 after treatment. Brains were quickly removed and were cut into 5 coronal sections, 2 mm thick. The sections were immersed in a 2% solution of 2,3,5-triphenyltetrazolium chloride (TTC; Sigma) in saline for 20 min at 37°C and then fixed with 4% paraformaldehyde. The infarct area was measured in each slice using computerized planimetry (PC-based image tools software). The infarct volume (mm^3^) was calculated as 2 mm (thickness of each slice) × (sum of infarct area in all brain slices).

### Immunohistochemistry and immunofluorescence confocal microscopy

At 1, 3 and 7 days after pMCAO, rats (n=6/experiment subgroup/time-point) were deeply anesthetized with 5% isoflurane and perfused intracardially with saline and 5% buffered formaldehyde. Brains were paraffin-embedded and sliced into coronal sections (5 μm) starting at 3 mm posterior to the anterior pole. Parallel sections were used to stain individually for the following primary antibodies: goat polyclonal antibody to CD11b (1:200; Santa Cruz Biotechnology, Inc., Santa Cruz, CA), mouse monoclonal antibody to NeuN (1:1,000; Sigma). Following extensive rinsing steps in 0.1 M PBS, the sections were reincubated in biotinylated rabbit anti-goat or goat anti-mouse immunoglobulin (Boster Biological Technology, Ltd., Wuhan, China) for 1 h at room temperature and then the Vector ABC system followed. For negative controls, the primary antibody was omitted. The positive cells of the 3 visual fields in the peri-necrotic cortex were calculated at ×400 magnification (one visual field=0.196 mm^2^).

For double immunofluorescence, the sections were incubated with a mixture of goat polyclonal antibody to CD11b (1:200) and mouse polyclonal antibody to GDNF (1:100), or with a mixture of goat polyclonal antibody to CD11b (1:200) and mouse monoclonal antibody to TNF-α (1:100) (all were from Santa Cruz Biotechnology, Inc.) overnight at 4°C, followed by a mixture of FITC-conjugated donkey anti-goat IgG and Cy3-conjugated donkey anti-mouse IgG (1:100; Jackson Laboratory, Bar Harbor, ME, USA). The sections were washed in TBS (0.2% Triton X-100 PBS) and mounted with Vectashield mounting medium (Vector Laboratories, Burlingame, CA, USA). Confocal images were captured by an Olympus FV300 confocal spectral microscope (Olympus, Tokyo, Japan) at ×200 magnification (one visual field=0.392 mm^2^). Image analysis was performed with ImageJ 1.39u (National Institutes of Health, Bethesda, MD, USA). The number of cells showing double immunostaining was estimated by counting cells in 3 random fields of the peri-necrotic cortex.

### RT-PCR

After evaluation of neurological deficits, rats (n=6/experiment group/time-point) were sacrificed with an overdose of sodium pentobarbital (75 mg/kg). Total RNA was extracted from the peri-necrotic cortex with the TRIzol kit (Fermentas Inc., Glen Burnie, MD, USA). Primers were designed with primer premier 5.0 software (Premier Biosoft International, Palo Alto, CA, USA) for CD11b (NP_036843/351bp), GDNF (NM_019139/206bp) and TNF-α (NM_012675/255bp) and β-actin (NM_031144/341bp). RT-PCR experiments followed standard protocols. Briefly, the cDNA strand first was synthesized from the total RNA and then the mixture of the primers and the cDNA products was amplified by PCR. Finally, the PCR products were separated on 0.8% agarose gel. Analysis was carried out using a gel imaging system (Bio-Rad Laboratories Inc., Hercules, CA, USA). Optical density (OD) of CD11b, GDNF and TNF-α was measured by the electrophoresis gel imaging system and their expression levels were calculated as percentage relative to β-actin control (OD of these proteins/OD of β-actin).

### Western blotting

The total sample adjacent necrotic cortex was dissociated from the rats which were sacrificed with an overdose of sodium pentobarbital (75 mg/kg) at 1, 3 and 7 days after treatment (n=6/experiment group/time-point). Brain tissues were lysed in RIPA lysis buffer, and the lysates were harvested by centrifugation (12,000 rpm) at 4°C for 30 min. Next, the protein samples (20 μg) were separated by electrophoresis in a 12% sodium dodecyl sulfate polyacrylamide gel and were transferred onto a polyvinylidene difluoride (PVDF) membrane. The membrane was placed in 5% non-fat milk for 1 h to block the nonspecific binding sites and was then incubated with the mouse polyclonal antibody anti-CD11b (1:1,000) or GDNF (1:1,000), or TNF-α (1:1,000). Then, the membranes were incubated with horseradish peroxidase-conjugated goat anti-mouse antibody (1:5,000) (all were from Santa Cruz Biotechnology, Inc.) at 37°C for 60 min. After four washes, the bands were detected with the enhanced chemiluminescence reagent (Cell Signaling Technology, Danvers, MA, USA). Band density was measured with ImageJ software (National Institutes of Health) and was standardized to that of GAPDH detected using mouse anti-rat GAPDH monoclonal antibody (Hangzhou GoodHere, Hangzhou, China).

### Statistical analysis

Observers blinded to experimental conditions evaluated outcome measures. A repeated measurement ANOVA was employed to analyze the behavioral measurement data. Student's t-test was used when comparing 2 groups. Differences were considered statistically significant when P-values were <0.05. All data are expressed as the means ± SEM.

## Results

### Measurement of neurological deficits

**Figure 1:**
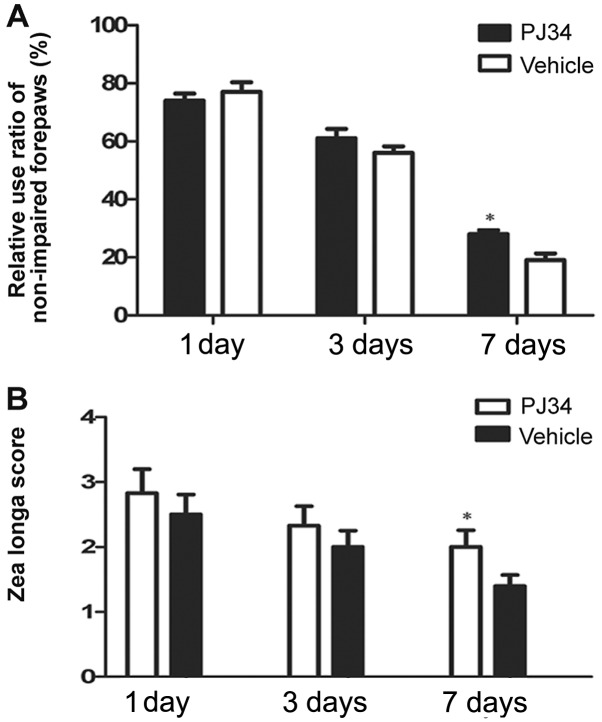
Evaluation of neurological deficits at 1, 3 and 7 days after treatment. (A) The cylinder test. Compared with the vehicle-treated rats, the PJ34-treated rats showed a larger relative percentage of using the non-impaired forepaw (left forepaws). A significant difference between the vehicle- and the PJ34-treated groups is noted at 7 days after the stroke; ^*^P<0.05 vs. the vehicle group. (B) The Zea Longa test. The PJ34 rats demonstrated a higher grade score than the vehicle rats at 7 days after the operation; ^*^P<0.05 vs. the vehicle group.

Neurological deficits were evaluated by the cylinder and the Zea Longa test at 1, 3 and 7 days following pMCAO. In the cylinder test, the PJ34-treated rats used the hemiplegic forepaws less than the vehicle-treated rats at 3 and 7 days after treatment. On Day 7 post surgery, there was a significant difference between the PJ34- and vehicle-treated rats in the frequencies of using the impaired forepaws (F=4.89, P=0.033) (Fig. 1A). In the Zea Longa test, the rats treated with PJ34 showed higher grade score than the rats treated with vehicle at 3 and 7 days post treatment. The difference between the two groups was significant in the grade score on Day 7 (F=7.50, P=0.021) (Fig. 1B).

### Lack of activated microglia increases infarct volume

**Figure 2:**
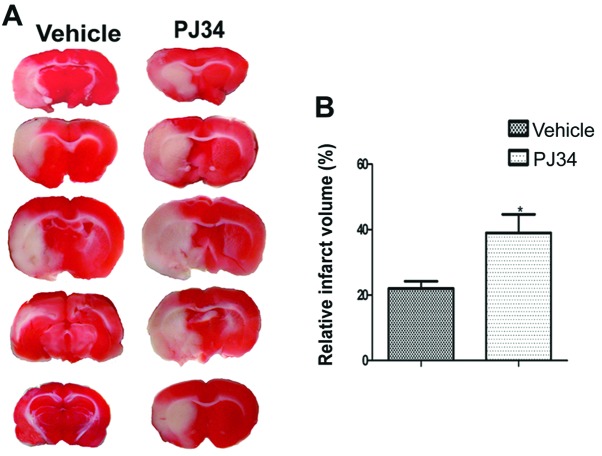
Infarct volumes of the PJ34- and vehicle-treated rats by TTC on Day 7 after treatment. (A) The infarct volumes of the PJ34- and vehicle-treated groups. (B) A significant difference between the two groups is observed in infarct volume; ^*^P<0.05 vs. the vehicle group.

The infarct volumes of the PJ34-and vehicle-treated rats (n=6/experiment group) were detected by TTC on Day 7 after the operation. Compared with the vehicle-treated rats, the PJ34-treated rats had a larger infarct volume (t=1.730, P=0.032)(Fig. 2).

### Greater neuronal loss in the absence of activated microglia

**Figure 3:**
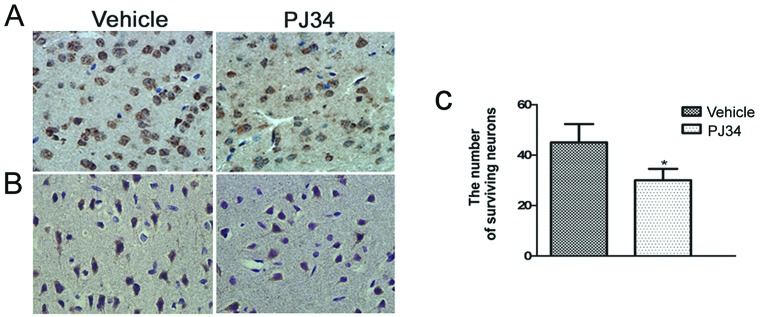
CD11b and NeuN immunoreactivity at 7 days after pMCAO. (A) CD11b immunoreactivity in the brain of the PJ34-treated rats is markedly attenuated compared with the vehicle-treated rats (x400); ^*^P<0.05 vs. the vehicle group. (B) The PJ34-treated rats (right) show fewer NeuN-positive cells in the peri-necrotic cortex than the vehicle-treated rats (left), (x400). (C) There was a significant difference in the number of NeuN staining cells between the PJ34- and vehicle-treated rats; ^*^P<0.05 vs. the vehicle group.

Immunohistochemistry was used to detect the CD11b- and NeuN-positive cells in the peri-necrotic cortex of pMCAO rats (n=6/experiment subgroup/time-point) at 1, 3 and 7 days following treatment. The number of CD11b- and NeuN-positive cells in the brain of the PJ34-treated rats was similar to the vehicle-treated rats at 1 day after treatment. Compared with the vehicle-treated rats, the number of CD11b- and NeuN-positive cells of the PJ34-treated rats was reduced on Day 3 and was reduced further on Day 7. The difference between the PJ34- and vehicle-treated rats was significant in the number of the CD11b-positive cells (11.4±1.1 and 21.6±1.3/field, respectively; t=2.471, P<0.01) (Fig. 3A) and the number of the NeuN-positive cells (t=2.283, P=0.029) (Fig. 3B and C) on Day 7.

### Expression of GDNF and TNF-α in activated microglia after SCI

**Figure 4:**
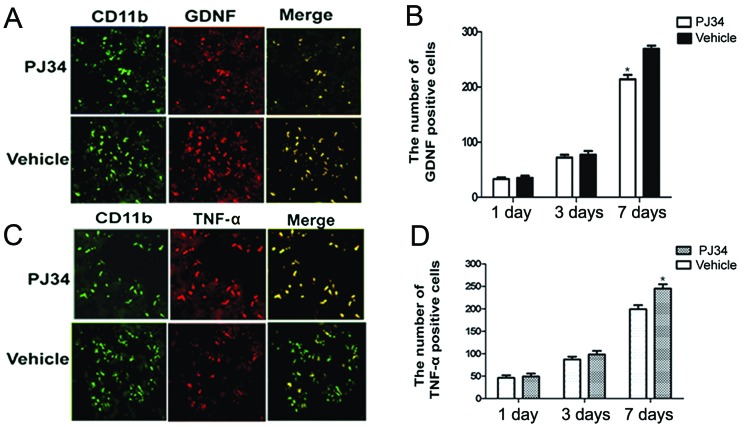
Co-localization of CD11b/GDNF and CD11b/TNF-α immunoreactivity on Day 7 after treatment. (A and C) On Day 7 after treatment, the PJ34-treated rats showed fewer CD11b (green)- and GDNF (red)-positive cells and double-labelled cells (yellow) for both CD11b and GDNF signals (A), but more TNF-α (red)-positive cells and double-labelled cells (yellow) for both CD11b and TNF-α signals (C) than that of the vehicle-treated rats (x200). (B and D) Change between the PJ34- and vehicle-treated groups in the number of GDNF- (B) and TNF-α-positive (D) cells at 1, 3 and 7 days after treatment. There were significant differences between the PJ34- and vehicle-treated rats with respect to the number of GDNF- and TNF-α-positive cells; ^*^P<0.05 vs. the vehicle group.

The GDNF- and TNF-α-expressions in activated microglia in the peri-necrotic cortex of rats treated with PJ34 or vehicle (n=6/experiment subgroup/time-point) were examined by double immunofluorescence at 1, 3 and 7 days after treatment. There was no difference between the PJ34- and vehicle-treated rats with respect to the expression of GDNF and TNF-α at 1 day after the treatment. Compared with the vehicle-treated rats, the PJ34-treated rats showed a reduction in GDNF expression in activated microglia at 3 days after treatment and a further reduction at 7 days (t=2.105, P=0.043) (Fig. 4A and B).

Although the total number of CD11b-positive cells in the brains of PJ34-treated rats was reduced at 3 and 7 days after treatment, TNF-α expression in the remaining CD11b-positive cells was increased at 3 days after treatment and further increased at 7 days relative to the TNF-α expression in CD11b-positive cells in the brains of vehicle-treated rats (t=3.741, P<0.05) (Fig. 4C and D).

### Inhibition of microglial activation influences the expression of the GDNF and TNF-α mRNA

**Figure 5:**
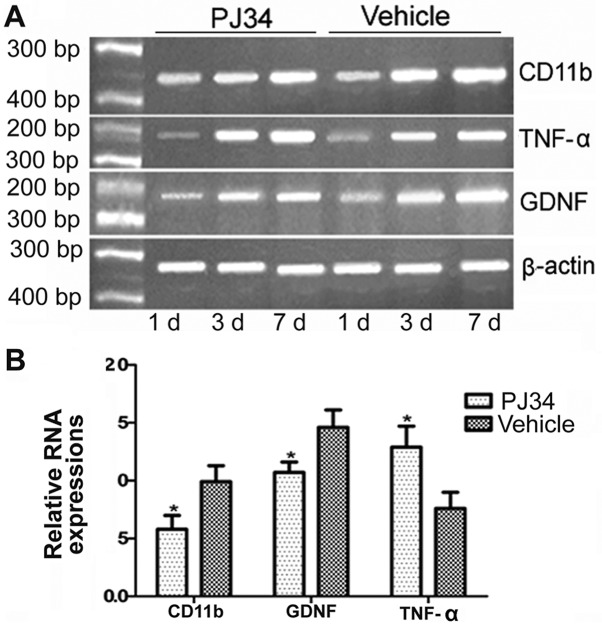
The mRNA expression of CD11, GDNF and TNF-α. (A) The expression of CD11, GDNF and TNF-α mRNA at 1, 3 and 7 days after treatment. (B) There was a significant difference between the PJ34- and vehicle-treated rats with respect to the expression levels of CD11, GDNF and TNF-α on Day 7; ^*^P<0.05 vs. the vehicle group.

The expression of the CD11b, GDNF and TNF-α mRNAs (n=6/group/time-point) was detected at 1, 3 and 7 days after treatment. There was no difference between of the PJ34- and vehicle-treated rats in the levels of CD11b, GDNF and TNF-α mRNAs on Day 1. The mRNA level of CD11b and GDNF was decreased on Day 3 after treatment and further decreased on Day 7 in the brains of the PJ34-treated rats compared with the vehicle-treated rats. However, the mRNA expression of TNF-α was increased on Day 3 after pMCAO and further increased on Day 7 in the brains of the PJ34-treated rats compared with the vehicle-treated rats (P<0.05) (Fig. 5).

### Inhibition of microglia activation influences the expression of the GDNF and TNF-α proteins

**Figure 6:**
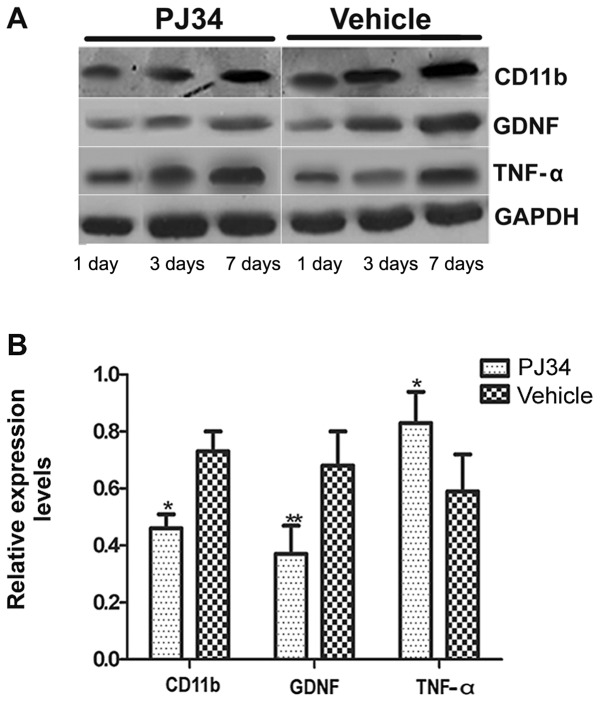
The expression levels of CD11, GDNF and TNF-α protein at 1, 3 and 7 days after treatment. (A) The expressions of CD11b, GDNF and TNF-α proteins in the brain of the PJ34- and the vehicle-treated rats at 1, 3 and 7 days after treatment. (B) There were significant differences between the two groups in the GDNF and TNF-α expression levels on Day 7 after treatment; ^*^P<0.05 and ^**^P<0.01, respectively vs. the vehicle group.

Western blotting (n=6/group/time-point) was used to determine the expression level of CD11b, GDNF and TNF-α proteins around lesion cortex of rats treated with vehicle or PJ34 at 1, 3 and 7 days following pMCAO. There was no difference between the PJ34- and vehicle-treated rats in the expression levels of CD11b, GDNF and TNF-α at 1 day after treatment. However, on Days 3 and 7 after the operation, the rats treated with PJ34 showed that accompanied by a decrease in the CD11b expression, GDNF expression was decreased but TNF-α expression was increased (Fig. 6A). There was a significant difference between the PJ34- and vehicle-treated groups with respect to the expression of CD11b, GDNF and TNF-α on Day 7 following treatment (P<0.05) (Fig. 6B).

## Discussion

The present study showed for the first time that activated microglia provide a neuroprotective role through balancing the GDNF and the TNF-α expressions following SCI. Compared with the vehicle-treated rats, the PJ34-treated rats had more severe neuronal deficits and a larger infarct volume and exhibited fewer activated microglia, greater neuronal loss, decreased GDNF expression and increased TNF-α expression at 3 and 7 days following ischemic stroke.

Currently, most findings related to the role of activated microglia in central nervous system disease (CND) are derived from *in vitro* analysis or a chronic neurodegenerative disease model (13). Previous data suggested that the different microenvironments inducing microglial activation lead to the different roles played by microglia in CND (14–16) while the condition after SCI is significantly different from the microenvironments in chronic neurodegenerative disease or the *in vitro* model (17). Therefore, *in vivo* investigations contribute more to knowledge of the effects of microglia after SCI. We hypothesized that the complete depletion of activated microglia is impossible *in vivo*. Thus, it is preferable to select an inhibitor to suppress the microglial activation as a control. Previous studies have shown that microglial activation correlates with PARP-1 which is an important co-activating factor and plays a crucial role in the nuclear factor (NF)-κB signaling pathway (18–20). Therefore, the inhibition of PARP-1 activity or NF-κB-dependent gene transcription by the inhibitor of PARP-1 blocks the signaling paths related to microglial activation (18,21,22) and hence microglial activation is suppressed (23,24). In addition, it has been well documented that PJ34, a PARP inhibitor, can effectively inhibit microglial activation (8,25,26).

To validate the inhibitive effect of PJ34 on microglial activation, we used immunohistochemical staining, RT-PCR and western blotting to examine the CD11b immunoreactivity and expression levels in the peri-necrotic cortex of the PJ34- and vehicle-treated rats post ischemia. Indeed, we observed that the CD11b positive cells were markedly reduced and the CD11b expression level was markedly decreased in the brains of rats treated with PJ34 compared with that of rats treated with vehicle 7 days after treatment. The results suggest that PJ34 can effectively inhibit microglial activation following brain injury.

We next considered whether activated microglia play a neuroprotective role after ischemic stroke. To address this, we compared the changes in motor function, the infarct volume and neuronal survival under the conditions of microglial activation or inhibition at 1, 3 and 7 days following treatment. The present results demonstrated that compared with the vehicle-treated rats, PJ34-treated rats had more severe neurological deficits, a larger infarct volume and exhibited fewer CD11b positive cells and greater neuronal loss on Days 3 and 7 after treatment. The lack of activated microglia in the penumbra regions leads to greater neuron loss and the enhancement of infarct volume. Therefore, there were more severe neurological deficits. These results indicate that activated microglia rescue the neurons in the penumbra area after SCI.

To explore the neuroprotective mechanisms of microglia, we detected GDNF and TNF-α immunoreactivity and their expression levels at 1, 3 and 7 days after surgery. GDNF and TNF-α are often investigated as they are two of the primary cytokines related to brain injury. Previous data have revealed that GDNF provides a potent neuroprotective role in animal models of Parkinson's disease (27), cerebral ischemia (28) and motor neuron degeneration (29). *In vitro* studies have shown that the phosphorylated extracellular signal-regulated kinase (ERK) is present in activated microglia (30). Previous investigations revealed that GDNF expression is associated with the phosphorylation of ERK (31). In the present study, we found that, along with the reduction in CD11b positive cells or the decrease in CD11b expression level, the number of GDNF positive cells and the expression levels of GDNF mRNA and protein were decreased at 3 and 7 days after treatment. These results indicate that the inhibition of microglial activation attenuates GDNF production. With regard to the mechanisms underlying the role of microglial modulation in the GDNF secretion, we speculated that reacted microglia induce the phosphorylation of ERK and further promote the GDNF secretion. However, the inhibition of microglial activation attenuates the phosphorylation level of ERK and the GDNF secretion is reduced further.

TNF-α is often considered a neurotoxic pro-inflammatory factor. Previous *in vitro* studies have demonstrated that upon stimulation with LPS, BV2 cells become overactivated and largely express the TNF-α protein (32). Contrary to these findings, our results indicated that in the absence of the activation of microglia, the number of TNF-α positive cells and the expression levels of TNF-α mRNA and protein were markedly increased. With regard to the regulating role of microglia in the TNF-α secretion, recent studies have revealed that the activation of P2X receptor (purinergic receptor) in activated microglia induced by ATP leads to the reduction of TNF-α release (33) while astrocytes can release ATP after brain injury (34). We inferred that activated microglia protect astrocytes (35) and promote ATP release from astrocytes after SCI. Subsequently, the increase of extracellular ATP activates P2X receptor of microglia and TNF-α secretion from microglia is decreased. However, when the activation of microglia is inhibited after SCI, the number of dead astrocytes is increased. Thus, ATP release is also reduced accompanied by the inhibition of P2X receptor activation. Therefore, TNF-α expression in microglia is also increased. Moreover, recent data have indicated that the presence of CD4^+^ T cells at sites of motoneuron injury in mSOD1 mice shifts the balance of microglial response from decreased GNDF expression and increased TNF-α expression to increased GDNF expression and decreased TNF-α expression after inherited amyotrophic lateral sclerosis (ALS) (36). However, further studies are required to ascertain whether the same role of CD4^+^ T cells is present in the brain of SCI.

We do not completely exclude the possibility that PJ34 affects the present results. However, several previous studies demonstrated that PJ34 provides neuroprotection after cerebral insult (8). Moreover, the results from double immunofluorescence and western blotting showed that the alterations of microglial activation are accompanied by the changes in GDNF and TNF-α expression levels. The time consistency between microglial activation and the secretions of GDNF and TNF-α further verifies that the present results are mainly attributed to the role of activated microglia rather than to PJ34.

In summary, the present study investigated the effects of activated microglia after SCI for the first time. Our results demonstrate that activated microglia exert a neuroprotective role through balancing the expressions of GDNF and TNF-α after SCI. These data suggest that microglia may be a potential target and vehicle for cerebral ischemic stroke therapy.

## Abbreviations

SCI: subacute cerebral ischemia
CND: central nervous disease
GDNF: glial cell line-derived neurotrophic factor
TNF-α: tumor necrosis factor-α
pMCAO: permanent middle cerebral artery occlusion
